# Maintaining the Balance of Intestinal Flora through the Diet: Effective Prevention of Illness

**DOI:** 10.3390/foods10102312

**Published:** 2021-09-29

**Authors:** Li Zhang, Zhenying Zhang, Lei Xu, Xin Zhang

**Affiliations:** 1 Department of Physical Education, China University of Mining and Technology, Beijing 100083, China; zhangli304036@126.com (L.Z.); zhangyucheng0603@163.com (Z.Z.); 2 Department of Food Science and Engineering, Ningbo University, Ningbo 315211, China; xuleinbu@163.com

**Keywords:** gut microbiome, brain–gut–microbial axis, dietary, mental illness

## Abstract

The human body is home to a complex community of dynamic equilibrium microbiota, including bacteria, fungi, parasites, and viruses. It is known that the gut microbiome plays a crucial role in regulating innate and adaptive immune responses, intestinal peristalsis, intestinal barrier homeostasis, nutrient uptake, and fat distribution. The complex relationship between the host and microbiome suggests that when this relationship is out of balance, the microbiome may contribute to disease development. The brain–gut–microbial axis is composed of many signal molecules, gastrointestinal mucosal cells, the vagus nerve, and blood–brain barrier, which plays an essential role in developing many diseases. The microbiome can influence the central nervous system function through the brain–gut axis; the central nervous system can also affect the composition and partial functions of the gut microbiome in the same way. Different dietary patterns, specific dietary components, and functional dietary factors can significantly affect intestinal flora’s structure, composition, and function, thereby affecting human health. Based on the above, this paper reviewed the relationship between diet, intestinal flora, and human health, and the strategies to prevent mental illness through the dietary modification of intestinal microorganisms.

## 1. Introduction

The maladjustment of intestinal microecology is the fundamental cause of many diseases, including the cardiovascular system and nervous system conditions, which are directly related to gastrointestinal disease [1]. The enteric nervous system (ENS) and the central nervous system (CNS) are connected through a series of intermediate neural pathways. Changes in the intestinal microecology can affect the activities of the CNS through the ENS, thus participating in the regulation of neural function, such as psychological, behavioral, cognitive, emotional, and neurological diseases [2]. The imbalance of the intestinal micro-ecological environment is closely related to Alzheimer’s disease (AD), Parkinson’s disease (PD), depression, and other neurological disorders [3].

A growing number of studies have found that gut microbiota may play an essential role in the prevention and treatment of depression [4]. The microbiome hypothesis describes the complex network structure of intestinal mucosa and the host-microbiome of depression patients, suggesting that depression is closely related to the intestinal microbiome, and that “gut microbiome–gut–brain” dysfunction is the primary pathological basis of depression. The abnormalities in this link may be the direct cause and potential influencing factors of depression, so the regulation of the microbiome may be an effective way to prevent and treat depression [5].

The sensory nerves of the gastrointestinal tract enter the central nervous system through the vagus nerve, the visceral nerve, and the pelvic nerve, so neurological disorders and PD are closely related to the balance of gastrointestinal flora [6]. In the nonmovement symptoms of PD patients, digestive system symptoms are especially prominent. For instance, many Parkinson’s disease patients are accompanied by gastrointestinal symptoms such as gastrointestinal peristalsis, loss of appetite, and constipation [7]. The behavioral changes caused by a high-fat diet may be due to gut microbiota, which in turn alter cognitive capacity [8].

Diet plays a vital role in regulating the composition and metabolic activity of human intestinal microflora [9]. Dietary nutrition contains substrates for the metabolism of intestinal microflora, which affect the design and function of intestinal microflora in many ways [10]. The source, chemical composition, physical and chemical properties, polymerization degree, and dosage of dietary fiber have different effects on the design of intestinal flora and fermentation products [11]. Understanding the relationship between dietary fiber composition and colonic bacteria is conducive to effectively maintaining or improving intestinal flora, and to preventing and treating mental diseases [12]. Based on this, the main objective of this paper was to provide new ideas for the further control and prevention of mental illness by diet.

## 2. Gut Flora and Brain–Gut Axis

The intestinal microflora plays an essential role in maintaining the stability of the intestinal environment, and its ecological imbalance is closely related to chronic intestinal inflammation, which can promote the development of chronic intestinal diseases and brain diseases [13].

### 2.1. Gut Flora

The gut is the body’s vast micro-ecosystem and the microbiome coevolved with its host to form mutual relationships. The maladjustment of intestinal flora can cause many diseases, and the imbalance of flora interacts with the disease [14]. The brain may regulate gastrointestinal function through the immune system, the nervous system, and the endocrine system. The gut microbiota may affect brain function through the vagus nerve, neurotransmitters (such as 5-hydroxytryptamine and dopamine), and neurotrophin (such as brain-derived neurotrophic factor) [15]. Therefore, a new biomedical concept, “Microorganism–intestine–brain axis,” was born. The intestinal microflora plays a crucial role in the interaction between the gastrointestinal environment and the CNS, affecting the functioning of CNS and the development of related diseases [16]. Fecal microflora mainly includes *Bacteroides*, *Prevotella*, and *Deinococcus*. Intestinal flora cannot only help the body to decompose and store fat, but also regulate immune function and promote angiogenesis. In addition, intestinal microflora can regulate immunity, endocrine, metabolism, and nerve function through immunity, neuroendocrine, and the vagus nerve [17]. Many patients with anxiety or depression are often accompanied by gastrointestinal dysfunction.

The host–gut microbiome interaction, genetic factors, immunity, and host environment can affect the composition of the intestinal microbiome, which in turn affects the host’s immune status and physiological function. Intestinal microflora participates in physiological processes such as nutrition metabolism, immune system development, behavior pattern formation, and stress response [18]. The stability of intestinal microflora and the existence of dominant bacteria are crucial for maintaining the normal physiological functions of the intestine. The absence or disproportion of specific bacterial strains results in various physiological abnormalities and diseases.

### 2.2. Bi-Directional Regulation Mechanism of Brain–Gut Axis

Intestinal microflora plays a vital role in regulating brain development and function, so some scholars believe that it is the “second genome” of the human body [19]. The gut has a complex two-way regulation with the central nervous system, known as the brain–gut axis or gut–brain axis [20]. The core of the “brain–gut axis” concept is the interaction between the gut flora and the central nervous system, hence the name “brain–gut axis” [21].

Because of this two-way feedback system, the brain can influence gastrointestinal function (such as movement, secretion, and mucin production) and immune function [22]. Similarly, emotional factors, such as stress or depression, can influence the development of chronic gastrointestinal diseases, such as inflammatory bowel disease (the most common being Crohn’s disease and ulcerative colitis) and irritable bowel syndrome [23].

Intestinal nerves are made up of a large number of neurons distributed in the gastrointestinal wall. Through the vagus nerve, its afferent neurons transmit small changes in the gastrointestinal tract to brain immune cells. Brain immune cells release neuroendocrine hormones that are critical to the host’s inflammatory and infectious response, such as cortisol, which works by altering intestinal permeability and barrier function, and by communicating with immune cells and cytokines [24]. Neurons and biochemical signals are transmitted throughout the body, including the pathways that are built up between the intestinal mucosa and the central nervous system, so the central nervous system is the hub of the brain–gut axis [25].

### 2.3. Gut–Brain Axis Influences the Distribution of Intestinal Microflora

There are more than 1000 species of symbiotic bacteria in the intestinal tract, which are divided into many species [26]. The gut–brain axis is an important bridge between the brain and the gastrointestinal tract. It can change the movement pattern of the gut by activating the immune system and further affect the composition of microbiota. It was found that stress could also affect intestinal motility, mucosal permeability, and the release of neurotransmitters and hormones, thus directly or indirectly altering intestinal microflora [27].

It has been found that the stress of mother–infant separation can change the structure of intestinal microflora in offspring rats, increasing the levels of tnf-α and ifn-γ in offspring rats, which further affects the system of the gut microbiome. It has been reported that the mice model of chronic stress showed marked depressive behavior, accompanied by increased brain neuroactivity, gut immune activation, and structural changes in gut microbiota. The above research indicated that the gut–brain axis could affect the distribution of intestinal microflora, thus further affecting the daily activities of the body [28].

## 3. Gut Flora and Mental Illness

The occurrence of functional bowel disease, including irritable bowel syndrome (IBS), is often affected by the imbalance of the brain–gut axis. Now, more studies have revealed that the alteration of microbial–gastrointestinal–brain axis regulation is likely to be involved in the development of autism, PD, emotional and behavioral disorders, and chronic pain, which is a potential pathophysiological mechanism of brain dysfunction and central nervous system disease [29,30].

### 3.1. Dual Relationship between Intestinal Microflora and Depression

The dysregulation of gut microbiota may contribute to depression by altering inflammatory cytokines, monoamine neurotransmitters, and brain-derived neurotrophic factors. Depression is associated with psychological problems that can cause changes in the structure of the gut microbiome [31]. Depression is one of the most well-known mental and psychological diseases, and is influenced by intestinal microflora changes [32]. Depression involves hypothalamus–pituitary–adrenal function, the monoamine neurotransmitter and its receptors, and related hormone levels, which is associated with changes in the gut microbiota [33]. Depression also involves changes in inflammatory cytokine levels [34], which may also be caused by changes in microbial flora [35].

Jiang et al. [36] found the intestinal microflora of patients with depression was significantly different from that of healthy individuals. At the level of species, the proportion of *Bacteroidetes*, *Proteobacteria*, and *Actinobacteria* in the intestinal flora of patients with depression increased significantly, and the proportion of *Firmicutes* decreased significantly. However, Lin et al. [37] found that patients with depression had more *Firmicutes*, fewer *Bacteroidetes*, and more *Prevotella*, *Klebsiella*, *Streptococcus*, and *Clostridium* XI. There was a positive correlation between *Prevotella* and *Klebsiella* and the scores of the Hamilton rating scale for depression (HAMD). Averina et al. [38] also observed an increase in *Alistipes*’s abundance in the gut of depressed patients. *Alistipes* are indole-positive bacteria that affect the metabolism of tryptophan. Tryptophan, a precursor of serotonin, may disrupt serotonin metabolism in the gut by increasing the levels of *Alistipes*. These findings suggest that depression is closely related to the gut micro-ecosystem. The gut micro-ecosystem may play a role in the development of depression through the indirect action of microbial metabolism or the direct effects of the immune system activation and infection.

**Figure 1:**
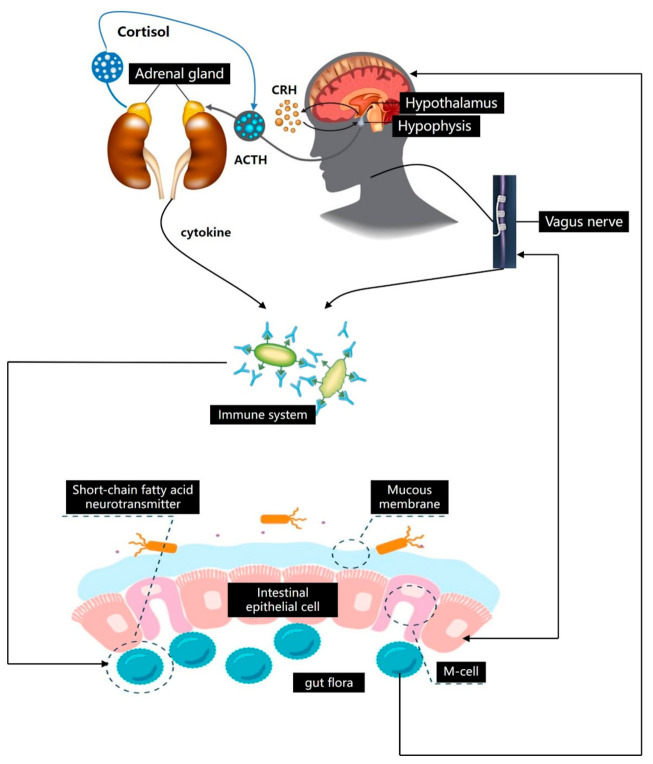
Microflora-regulation mechanism of brain–gut axis. The pathogenesis of depression mainly focuses on the hypothalamic–pituitary–adrenal axis, the monoamine neurotransmitter and receptors, the immune system, and the gut microbiota in the endocrine system.

There is much evidence that the imbalance of microbiota is closely related to depression and depression-related behaviors [39]. Studies have found that there is a significant microecological imbalance in the gut of patients with depression. The proportion of *Enterobacteriaceae* and inflammatory bacteria *Alistipes* increased significantly. In contrast, the proportion of anti-inflammatory bacteria *Faecalibacterium* decreased significantly [40]. These changes may be related to intestinal microflora translocation, immune system activation, and HPA hyperstimulation in depression [41]. At the same time, gut microbiota can affect neurotransmitters. Many bacteria can produce or secrete neurotransmitters, such as GABA produced by *Lactobacillus* and *Bifidobacterium* [42]. Studies have shown that GABA has a certain effect on the brain–gut axis and dysfunction of depression. In addition, microbial metabolism products, such as short-chain fatty acid and sodium butyrate, also affected depression [43]. Dinan et al. [44] found that, compared with healthy controls, the depressed patients’ gut microbiota extracts and butyrate metabolites were produced by relatively few bacteria, and sodium butyrate had an antidepressant effect. Therefore, the imbalance of gut microbiota as the second brain is the result of the regulation of the first brain [45], but it also may be involved in the development of depression through the brain–gut–microorganism axis (Figure 1).

### 3.2. Relationship between Intestinal Microflora and AD

AD is a primary neurodegenerative disease, also known as Alzheimer’s disease. Cognitive dysfunction, progressive memory loss, behavioral abnormalities, and social dysfunction can lead to chronic inflammation, brain damage, and neuronal death due to continuous microglial activation during aging [46]. In addition, the imbalance of the microbiota in the gut, mouth, and nasal cavity can lead to a systemic inflammatory response and activate a microglia immune response [47]. The activation of reactive gliosis can trigger astrocytes, further aggravate the neuroinflammation and blood–brain barrier (BBB) dysfunction [48], destroy the neurons, and enter a vicious circle. With the increase in age, BBB defenses weaken, so pathogens (viruses, bacteria, and fungi), immune cells, and their metabolites enter the brain [49]. Recent etiological studies of AD have suggested that inflammation-mediated BBB decay and unstable cortical maturation may depend on gut microbiota, thus supporting the hypothesis that gut microbiota drives chronic inflammation associated with neurodegenerative or neurodevelopmental diseases such as AD [50].

Recent studies have shown that the concentration of acetate in the intestine of AD Drosophila melanogaster is higher, but the proportion of acetate is severely downregulated [51]. Butyrate is a multifunctional molecule. Many of its effector cells can specifically bind to G protein-coupled receptors (FFAR3 and FFAR2), affect the functions of many genes and cell proteins, and have neuroprotective effects [52]. Butyrate is the effective agonist of FFAR3, while acetate and propionate are the agonists of FFAR2. Kilgore et al. found that butyrate had a significant effect on improving the learning and memory of AD mice, even in the latter stages of the disease [53].

### 3.3. Gut Flora and Parkinson

PD is the second-most common neurodegeneration in the world after Alzheimer’s disease. According to statistics, there are about 5.8 million Parkinson’s disease patients globally and about 2.6 million Parkinson’s disease patients in China, ranking first globally [54].

Gastrointestinal symptoms often precede the motor symptoms in PD patients, and the most common symptom is constipation [55]. The incidence of constipation in PD patients was 61.4%, and 24.5% of PD patients had constipation before the onset of motor symptoms [56]. If the motor and nonmotor symptoms are more severe, cognitive impairment, insomnia, and autonomic nervous symptoms will be more profound [57].

When intestinal microbiota decompose dietary fiber, they produce a variety of metabolites, including SCFAs ingested by the epithelial cells of the colon, which affect the physiological processes of the host [58]. Intestinal microflora promotes α-syn-mediated dyskinesia and brain-related pathological processes by producing SCFAs. Without the gut microbiota, SCFAs cannot be created, thus reducing the excitability of the microglia and limiting α-syn-mediated brain pathology [59]. SCFAs regulate the activity of microglia and promote the development of Parkinson’s disease [60]. The results showed that SCFAs-fed GF mice had the same dyskinesia as mice with normal gut microbiota, and there was an abnormal accumulation of α-syn in the PD-related brain region. The results suggest that SCFAs may activate central nervous system microglia, trigger a neuroinflammatory response, and lead to neuronal damage and even death [61].

## 4. Dietary Intervention: Improving Intestinal Microbiota Imbalance to Prevent Mental Illness

Intestinal microflora can affect the nutrition and energy balance of the host, and regulate the immune environment [62]. However, in some cases, the reciprocal relationship becomes skewed, with changes in diet and long-term exposure to antibiotics and other drugs [63]. The contribution of the host’s gut-balanced diet to microbial dysfunction and its vital role in coordinating host-microbiome interactions have been demonstrated [64].

### 4.1. Regulation of Dietary Fiber on Intestinal Flora: An Effective Method to Prevent Mental Disease

Dietary fiber’s physical and chemical properties include solubility, viscosity, particle size, absorbability, and water holding capacity, which are mainly related to dietary fiber’s molecular weight and structure [65]. The physiological characteristics of dietary fiber include lowering blood pressure, preventing heart disease, preventing stroke, controlling body weight, slowing gastrointestinal disorders, improving blood lipid level, controlling postprandial blood glucose, and improving immunity [66]. Dietary fiber intake usually changes the size and length of digestive organs such as the small intestine, cecum, and colon. This is related to the morphology of intestinal epithelium and ultimately affects the digestive and hydrolytic functions of the intestinal tract [67]. Dietary fiber is fermented in the intestine to produce butyrate, which has a positive effect on the intestinal epithelium. First, it is the preferred substrate for epithelial cells, and secondly, it can improve the intestinal barrier by reducing the concentration of local oxygen-inducible factors in the intestinal epithelium. Dietary fiber directly binds to Toll-like receptors, which are immune-regulating receptors. When dietary fiber is ingested, the immune defense system is more alert; however, due to the lack of effective human health or disease biomarkers, the relevance of these results remains uncertain [68].

**Figure 2:**
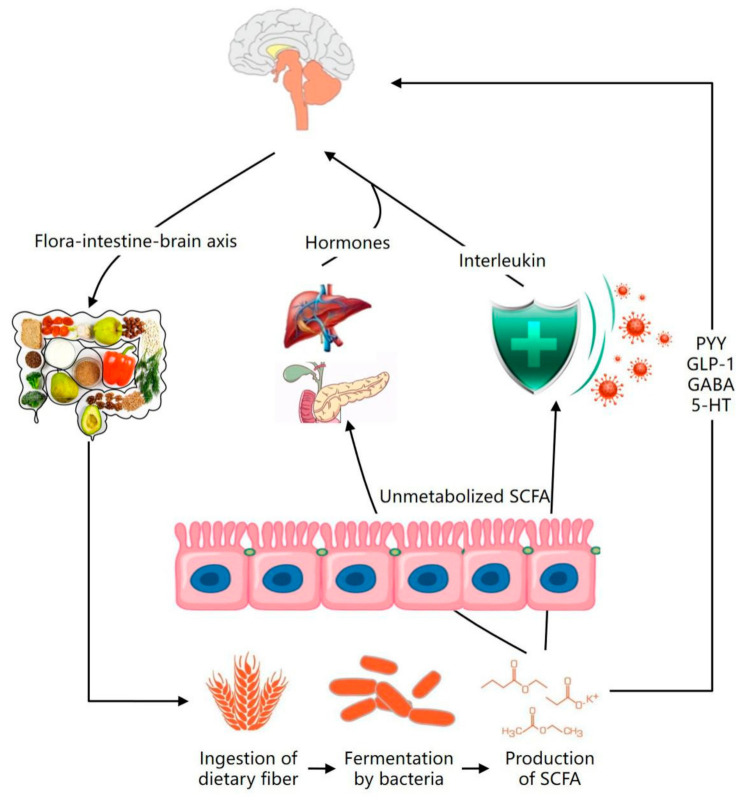
Dietary fiber affects gut microbiota to prevent mental illness. The circulating SCFAs are converted into hormones, 5-HT, GABA, GLP-1, and PYY by the immune system, hepatopancreatic portal system, and adipose tissue, thereby breaking through the blood–brain barrier into the blood–brain border of the central nervous system.

The physiological properties of dietary fiber include preventing heart disease, preventing stroke, controlling body weight, alleviating gastrointestinal disorders, and enhancing immunity. Dietary fiber produces a series of metabolites, the most important of which is SCFAs, estimated to be more than 90% absorbed by the gut or utilized by the gut microbiome [69]. As a signaling molecule, SCFAs are absorbed by intestinal epithelial cells and bind to two G protein-coupled receptors. 5-hydroxytryptamine (5-HT) is an important neurotransmitter involved in the regulation of gastrointestinal motility and secretion, can regulate intestinal permeability, promote intestinal peristalsis, and reduce the absorption time of food energy substances in the 5-hydroxytryptamine [70]. The circulating SCFAs are converted into hormones, interleukin, 5-HT, GABA, Glucagon-like peptide-1 (glucagon-like peptide-1, GLP-1), and peptide tyrosine (PYY) by the immune system, hepatopancreatic portal system, and adipose tissue [71]; break through the blood–brain barrier into the central nervous system; and play a broad regulatory role on the body through the influence of the gut–brain axis (Figure 2).

As a neurotransmitter of the peripheral nervous system, 5-HT exists widely in the gastrointestinal tract and is closely related to gastrointestinal diseases by binding to 5-HT-specific receptors to regulate gastrointestinal activity [72]. The study found that after taking 5-HT signaling system antidepressants in patients with depression, gastrointestinal disease has a certain degree of relief [73]. The content of 5-HT is decreased in patients with gastrointestinal motility disorder, but increasing 5-HT content could improve gastrointestinal function [74]. At the same time, depression-induced brain–intestinal axis dysfunction can affect gastrointestinal 5-HT secretion [75]. In vitro studies have confirmed that when the 5-HT receptor signaling pathway is abnormal, the esophageal smooth muscle relaxation response is weakened [76].

Resistant starch is a type of dietary fiber. It is defined as “starch and its degradation products that are not absorbed by the small intestine of healthy individuals.” Prebiotics has a symbiotic relationship with probiotics. As resistant starch can be fermented and used as a substrate by colonic microbiota, it can promote the growth and activity of probiotics, and interact with other probiotic dietary fiber to exert its probiotic effects [77]. The metabolism of intestinal microorganisms produces products such as SCFAs and lactic acid through wholly or partially fermented starch that cannot be hydrolyzed by enzymes. These starches contain more than 60% acetate, 69.5% butyric acid, 50.2% propionic acid, 44.1% valeric acid, 20.3% isovaleric acid, and 19.2% caproic acid. Resistant starch can produce butyric acid under the fermentation of intestinal microorganisms, and its ability to produce butyric acid is higher than other dietary fiber [78]. The intestinal and fecal SCFAs contents are increased, and the intestinal microflora of the host are changed adaptively after humans and animals eat foods with high resistant starch content.

Studies have shown that healthy foods are rich in dietary fiber, unsaturated fats, and fermented foods such as yogurt and cheese [79]. As they contain fewer refined carbohydrates, sugars, and food additives, eating these healthy foods can improve human health by increasing the diversity and stability of gut microbiota [80]. In addition, a healthy diet can improve behavior and cognition by enhancing the gut–brain axis, thereby stimulating the growth of beneficial bacteria [81]. The number of genes and the type of basal ganglia are different, indicating that the selective consumption of dietary fiber determines the preference of specific flora in the intestine, and affects the balance of strains in the colon. Therefore, dietary fiber can affect the composition of the intestinal microflora, thereby improving health.

### 4.2. Regulation of Tea Polyphenols on Intestinal Flora—Effective Prevention of Mental Diseases

Tea polyphenols (TPs) are the leading natural active ingredient in tea, which has been proven to have antioxidant activity, improve cognitive and learning memory, neuroprotection, and other pharmacological activities [82]. Phenol compounds in tea can inhibit the growth of intestinal pathogenic bacteria, create a good intestinal environment for the development of beneficial bacteria, and promote helpful bacteria’s physiological activities [83], thus promoting the physiological activity of beneficial bacteria and regulating the structure of intestinal flora. In addition, TPs can directly control the movement of certain intestinal microbial enzymes, which are mainly achieved by binding to enzyme protein molecules [84]. It was found that catechin binding at the ATP binding site of the protein subunit inhibited the activity of *E. coli* [85].

TPs have been shown to have broad-spectrum antimicrobial properties. A large number of studies have shown that TPs have potent inhibitory effects on 19 kinds of bacteria, such as *Bacillus subtilis*, *Staphylococcus aureus*, and *Salmonella*. Moreover, different bacteria have different resistances to TPs, which is related to the specific composition of TPs and the characteristics of bacteria [86]. The antibacterial activities of catechins, the main antibacterial substances in TPs, are also different. Tea polyphenols can significantly inhibit the growth of *E. coli*. TPs can destroy the cell structure of *E. coli*, increase the permeability of the cell wall, and reduce the number of *E. coli* [87].

There are many phenolic hydroxyl groups in the molecular structure of TPs. Most dietary polyphenols are transformed into metabolic derivatives with different forms and functions by intestinal microflora [88]. Therefore, the metabolism and absorption of TPs in the human body mainly depend on the transformation of intestinal microorganisms. The intestinal flora can hydrolyze glycosides, glucuronides, amides, esters, and lactones [89]. TPs also undergo a series of cyclic cleavage, reduction, demethylation, and dehydroxylation reactions. Once absorbed, the microbial breakdown products are absorbed into the portal vein and transported to the liver. They undergo the phase II binding reaction and produce glucuronic acid, sulfated and methylated conjugates, or their combinations [90]. These conjugates circulate in the blood, possibly pass through the liver and intestines, and are eventually excreted in urine or feces [91]. However, each individual’s microbial biotransformation ability affects the final metabolites and their bioavailability; each phenolic compound’s health effects on different populations stem from individual differences in gut microecology, which also determine the absorption of polyphenols.

A study showed that after three weeks of TPs intervention, the proportion of *Bacteroidetes* and chlorophytes increased, and the intestinal microecological disturbance caused by a high-fat diet was improved, which showed that TPs could significantly improve the microbial diversity in high-fat mice [92]. Studies on the effects of catechins on intestinal microflora have shown that methylated catechins promote the proliferation of beneficial bacteria such as lactic acid bacteria and *Bifidobacterium* in the gut, and effectively inhibit the expansion of *Bacteroides* and *Clostridium* [93]. It can be concluded that TPs can improve the diversity of intestinal flora, change the abundance of certain flora, and enable beneficial bacteria to better promote human health.

## 5. Conclusions

At present, a close relationship between intestinal microbiota and mental illnesses has been proven. Intestinal microbiota cause neuroendocrine and intestinal nerve disorders through the brain–gut axis, further causing the imbalance of inflammatory factors and metabolites. At the same time, mental illness can also cause intestinal microbial disturbance through changes in the brain–gut axis. In this article, we discussed the role of diet in improving gut microbiota in the prevention of mental illness. With the development of neuroscience and immunology, research will further reveal the key role of diet in preventing mental illness by regulating intestinal flora.

## Data Availability

The study did not report any data.
